# Interaction rituals, emotions, and early childhood science: digital microscopes and collective joy in a multilingual classroom

**DOI:** 10.1007/s11422-021-10056-6

**Published:** 2021-07-07

**Authors:** Sara E. D. Wilmes

**Affiliations:** Institute for Teaching and Learning, The University of Luxembourg, Esch‑sur‑Alzette, Luxembourg

**Keywords:** Interaction rituals, Early childhood science, Emotions, Multilingual, Plurilingual

## Abstract

In her original article, “Identity, Agency and the Internal Conversations of Science and Math Teachers Implementing instructional reforms in High-Need Urban Schools”, Stacy Olitsky (2021) takes us on an exploration of the identity development and agencies exerted by two teachers working to implement science instructional reforms in high-need urban schools. Olitsky (2021) utilizes Interaction Ritual Theory as a lens to examine a seldom viewed and even intimate aspect of teacher’s worlds, namely teachers’ self-talk. In this forum article I embrace the invitation extended by Olitsky, through an exploration of the interaction rituals that took place among students and a teacher working with digital microscopes in an early childhood classroom. I draw upon the theoretical lens of *communitas* to illuminate the power of collective joy that formed. Specifically, I will share two vignettes from a multilingual early childhood classroom to illustrate how teacher-guided and student-guided spaces afforded interactions that lead to the development of collective joy. I show how collective work with the microscopes allowed for joy and surprise to occur within a classroom of plurilingual students who are participating in their first schooled experiences of science. I conclude with a discussion of the power of student-driven instructional spaces as places for students working to learn science, and the language of instruction, to collectively experience joy as they explore.

In her article, “Identity, Agency and the Internal Conversations of Science and Math Teachers Implementing instructional reforms in High-Need Urban Schools”, Stacy Olitsky (2021) explores an intimate aspect of teacher’s professional lives: self-talk. Through an analysis of interaction rituals, Olitsky presents an examination of the self-talk of teachers within reflective journals and relates this to their implementation of instructional reform in high-need urban schools. I embrace the invitation extended by Olitsky (2021), and in this forum article I delve into the emotional spaces formed in a multilingual early childhood classroom engaged in science investigations. Through an elaboration of two vignettes developed using interaction ritual analysis, I show how the teacher’s use of an open science pedagogical approach that honors individual and collective work, created places in which the young students experienced collective joy as they engaged in science. This work contributes to a growing body of scholarly work that illuminates the power of interaction ritual analysis to reveal nuances of the emotional landscapes within classrooms, in this case with a classroom of young students who are working to master the language of instruction as they engage in science.

## Interaction rituals, emotions, and science education

In company with Olitsky (2021), there exist a growing number of scholars who have examined emotions in the context of science education. One analytical lens employed in this scholarly work, and used by Olitsky, draws upon the sociology of emotions, and relatedly, Interaction Ritual Theory (IRT) (Collins 2004). IRT and interaction ritual analysis involves an examination of emotion at micro-levels (tenths of a second) within social interactions. It is these small, almost imperceptible movements and expressions that develop through interaction that can lead to the buildup of collective emotion. IRT is grounded in an ontology that honors the role of the body and the material in interactions along with the cognitive, and the roles these facets play in the development of emotions that arise through interactions. As Hubbard (2007) explains, “Individuals are only able to express themselves in space through their bodies–corporeal physicality representing the basis of “being in the world” (p. 121). More often than not, how students and teachers' bodies interact through time and space, and how this corporality relates to engagement and meaning making is seen as secondary. Through the lens of IRT, however, we can embrace an embodied ontology and “interpret space as more than contextual: it is instead regarded as material the body engages and works *with*" (Lupton 1998; emphasis in Hubbard 2007, p. 121). Emotions arise as the body and mind interact with the material world, and with others, and are co-created over time and space through these interactions (Hubbard 2007).

In a study employing IRT and interaction ritual analysis, Catherine Milne and Tracey Otieno (2007) found that teacher science demonstrations can lead to a build up of collective student emotion grounded in classroom experiences. Relatedly, in previous work Olitsky (2007) explored how interaction rituals form on micro-levels in interaction between a teacher and students in a high school chemistry classroom that led to short-term collective emotion, at times positive, and at times negative. In our 2017 seminal paper, Christina Siry and I were able to bridge the use of IRT in science education to examine interactions in multilingual classroom interactions with plurilingual students. Our analysis revealed how primary students communicated by drawing upon communicative resources from several national languages, as well as employing bodily and material resources. Over time, micro-level interactions resulted in the buildup of positive emotions and synchrony among the students in ways that supported the inclusion of students who were less comfortable using the language of instruction. Collectively these studies, along with a growing body of scholarly work (e.g. Rinchen, Ritchie and Bellochi 2016), show how interaction ritual analysis can be used to examine the embodied and emotional aspects of interaction that play key roles in classroom engagement and interaction.

## Communitas: Collective joy

*Communitas* is a concept that refers to the collective joy that may develop among members of a community or group engaged in joint experience. First explained in-depth in academic circles by the Anthropologist Victor Turner (1969) in his writing, “Liminality and Communitas”, Turner developed his theorization of *communitas* as he drew upon the Latin concept of *communis* (meaning: common, joint, public, collective, social, shared, possessed or used by all (Oxford Latin Dictionary, 1982). Turner applied a lens of *communitas* to analyze and theorize what he was experiencing as he explored ritual interactions within diverse communities in Africa and South America. Through detailed observations and participation with communities in their rituals, Turner proposed *communitas* as a collective place of anti-structure, where structure is turned on its head. Communitas is a place of “spontaneous, immediate, concrete” emotional experience, which he positioned in direct opposition to the more norm-governed, institutionalized social structures” of many communities (Turner 1969, p. 372). *Communitas* differs from the concept of community. Community is often used to refer to people within a place (real or virtual), who feel a form of solidarity, and who are united through emotion for a cause. *Communitas*, in comparison, is an emotional state that arises suddenly and is emotionally all-encompassing. It is spontaneous and can arise in communities through joint experience. Through living with, getting to know, and being present in body with diverse communities, Turner experienced how rituals allow spaces for participants to be “released from structure into (anti-structure or) *communitas*” and in these places, to experience a common being, a common oneness that is free from social positioning, that allows participants to “return to structure revitalized by their experience of communitas” (Turner 1969, p. 373).

Edith Turner (2012), Victor’s partner, went on to extend this conceptualization after Victor's death in their collaborative ethnography, *Communitas: The Anthropology of Collective Joy*. In this work Edith expands the lens of *communitas* across a wide range of communities, rituals, and aspects of life including communities interacting in nature, at work, following natural disasters, acting in revolutions, and during national celebrations. In this more recent work, Turner (2012) deepens the theorization of collective emotions. *Communitas*, she explains, “appears unexpectedly in group action. It has to do with the sense felt by a group of people when their life together takes on full meaning.” Within communitas one is “freed from the regular structures of life” (p. 2) and experiences a collective closeness. Thus, *communitas* affords a lens on collective life that explores how collective action and emotion can allow for breaks or departures from the structures of social life. In this forum article, I explore how *communitas* forms in an early childhood classroom exploring with digital microscopes. I will present vignettes of interaction rituals that develop as the teacher and her plurilingual students explore. Through a presentation of the vignettes I explore the questions, *what rituals form through micro-level interactions in these spaces of exploration*? And *which emotions related to communintas / collective joy can be seen*?

Following a presentation of the vignettes that unpack these questions, I discuss how spaces were created that allowed for the development of collective joy. I then discuss implications of this work for research and for science instruction with children who are first experiencing science in school, and who are learning science through a language they are also working to learn.

## Methodology, participants and analytical approach

The analysis I present next was compiled utilizing interaction ritual analysis, grounded in IRT (Collins 2004). Analysis involved viewing video segments of classroom interaction at one-tenth of a second frames, and noting participants’ gaze, position, facial expression, and verbalizations relative to their interactions over time (Wilmes and Siry 2018). This led to a focus on two specific events, one teacher-guided and one student-guided. Next, I unpack the group emotions and interactional patterns that developed and describe these through a lens of *communitas* (Turner 2012). Data analyzed included videos, classroom artefacts, and interviews conducted by our research team within our project which supports the teaching of science in plurilingual primary classrooms in Luxembourg.

### Martine’s class and her plurilingual students

Martine is a teacher in a local primary school in Luxembourg. We have collaborated for several years now through our collective work at the SciTeach Center at the University of Luxembourg, a resource center dedicated to supporting science teaching in early childhood and primary schools (Siry, Andersen and Wilmes 2018). The SciTeach Center is a collaborative effort between researchers and teachers, and as a part of our collaboration to develop teacher professional development courses, Martine invited me and our center director Christina Siry to come to her classroom to work with her and her students. We decided that we would pilot two series of inquiry-based activities using digital microscopes, that we aimed to develop into teacher workshops to offer through the SciTeach Center.

Martine’s classroom is a magical space. It is filled with colors, sounds, and the imaginings of her four- and five-year-old students as apparent in their work that adorns the classroom walls, and amplified through the sound of their diverse voices. It is a space of action, and of talk, a space of silence, and of respect. Together, they form a community that works together through their diversity, to learn together. This is a primary multiage classroom of 4- and 5-year-old plurilingual students in their first and second mandatory years of schooling in Luxembourg. In our national context families may first enroll their children in an optional year of schooling at 3 years old. This is followed by mandatory kindergarten at 4 and 5 years old. These initial three years comprise the early childhood component of public education. The goal of these initial three years with respect to language is to provide students with access to and opportunities to learn and to socialize in and through Luxembourgish, one of our three national languages (Luxembourgish, French, German).

With regard to science, the Luxembourg national curriculum (Plan des Études, MENFP 2011) details competencies in *Decouverte du Monde* (Discovery of the World) that students at this level are to develop through engagement in science practices such as observing, investigating, and communicating about their science experiences. At the time I worked with Martine, her multi-aged class consisted of fifteen pupils in either their first or second year at this level. Martine explained that this year, as was common in past years in this school district, students represented nine different nationalities. Of the 15 students in the class, one spoke Luxembourgish, the language of instruction, at home with family. Additional languages spoken at home by students included Creole, Arabic, Chinese, Farsi, Portuguese, Italian, and German. In the research group I belong to, we employ a lens of *plurilingualism* to describe our students and to situate their communicative competencies. A *plurilingual* lens valorizes our students' ability to draw from and utilize diverse communicative resources from several national languages and through embodied and material ways of communicating. This contrasts with a *multilingual* lens, which refers to students as a language-learners, multilingual, or bilingual, and that situate students’ communicative competencies relative to an idealized national speaker who draws upon one national language (or multiple idealized national languages as whole entities). We use the term *multilingual* to describe spaces in which people employ their linguistic repertoires. This use of *plurilingual* to describe people, and *multilingual* to describe spaces is in accordance with the Council of Europe's (2018) position on plurilingualism. Thus, in our work we explore the resources plurilingual students in Luxembourg employ in multilingual classroom spaces (e.g., Wilmes and Siry 2021). Positioning students as plurilingual provides us with a lens on the diverse, multimodal resources they employ in classrooms (Wilmes, Siry, Gómez Fernández, Gorges 2018). 

Many of the students in Martine’s class were born in Luxembourg. A few had move to Luxembourg within the past few years as political refugees. There was one student with health issues related to a trauma. There was a student who was identified as being on the autism spectrum, and who had a support teacher who would shadow him for the day. This was a class of 15 vibrant souls, carrying different shades of being and histories in their bodies, and who brought these with them each day to this space of school to be with Martine. The diversity of Martine’s class is typical for Luxembourg. Currently in our Spillschoul classes (4- and 5-year-olds) approximately fifty percent of students hold nationalities other than from Luxembourg, and almost fifty percent speak a language other than the 3 national languages (Luxembourgish, French, German) at home (MENJE 2019). This diversity is reflected in our public schools. Martine’s students are exceptional, in that they represent their own unique histories and trajectories, and yet representative in that they reflect local and national levels of diversity.

I came to this class, to work with Martine, in order to co-develop lessons and science units to incorporate into the teacher professional development courses we offer through the SciTeach Center at our University. The data I share here were collected during our process of co-teaching and co-development. The data corpus consisted of classroom videos from whole class and tabletop cameras collected over a 4–6 week unit on Living Things: Worms and Compost. The use of digital microscopes was new for all of the student. Martine had experience using the microscope through her work with the SciTeach Center and using them when she led teacher professional development workshops for teachers within our national system. The multilayered analysis I share involved video analysis that zoomed into the micro-level to examine interactions and emotions as students worked with the digital microscopes, coupled with zooming out to examine classroom interactions layered with insights gathered from conversations with Martine as we co-taught. This allowed me to see how through interaction, and the material environment, interaction rituals developed that led to the production of *communitas*, or collective joy.

### Digital microscopes in early childhood science

Within the last decade, advances in technology and materials production have led to the development of small hand-held wireless, yet high-quality digital microscopes. While obtaining these easy-to-use digital tools is rather straightforward, a literature review revealed that only a handful of studies have explored their use in science instruction. MacGregor Kinseley and Karen Capraro (2013) provide a close-up view of several science investigations they conducted with students comparing the development of differ forms of insects raised in their classroom. Pietro Baroni and colleagues published a study in 2014 in which they examined the introduction of digital microscopes in early childhood classrooms in Italy. They found that the incorporation of the digital microscopes in science instruction increased student's enthusiasm and supported not only science, but also geometry and art instructional objectives.

Martine incorporated digital microscopes into her early-childhood classroom throughout the school year. For the Worms and Compost project at the center of the analysis I present here, Martine support students' initial use of the digital microscopes over several session of inquiry-investigations guided by their own questions. Students were able to engage in investigations either in pairs or individually, at laptop and digital microscope setups arranged around the classroom.

### Interaction ritual analysis

The vignettes I present next were constructed through a process of interaction ritual analysis, a similar process as utilized by Olitsky (2021). Through a micro-analytical view, interactions are viewed at a tenth of a second in succession. This provides insight into the movements, gestures, relative actions of people and materials engaged in a space as they participate in science. I conducted interaction analysis using a multilayered approach (Wilmes and Siry 2018). First, I constructed a video log (for 30 h of video) detailing the instructional steps undertaken during each science session. Then, I zoomed in to examine students’ microscope use, and constructed a log specific to the microscope’s use indicating who was involved in interaction, what they observed, and what was discussed. This provided a layer from which I was able to then zoom in further into the moment-by-moment frame-by-frame interactions (at one-tenth of one second). From this point, I then layered on information gathered through interviews  and co-teaching conversations with Martine, to contextualize what I was seeing on micro-levels. From this process, I produced vignettes of students’ interactions through which I was able to explore aspects relative to the formation of emotions including *communitas*, or collective joy.

## Vignettes: Emotions, bodies, spaces, and digital microscopes

In the sections that follow I present two vignettes, one from a teacher-guided interactional space, and one from a student-guided space, interwoven with interview excerpts, to detail collective action as the students worked with the digital microscopes.

### Vignette 1: Come look!

**Fig. 1:**
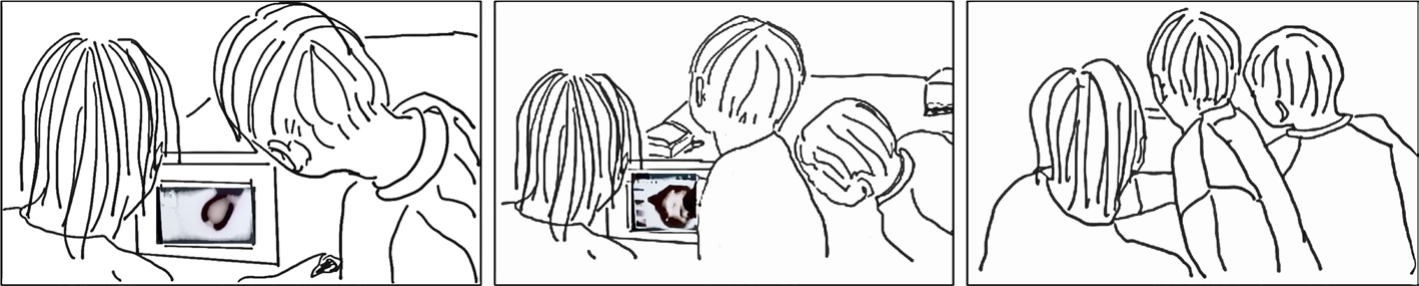
Martine and a student view a worm cocoon on the laptop using a digital microscope and are joined by a third student

The clear box of compost dirt and worms was set up in the middle of the student-level table at the far-end of the classroom. Students were able to stand at their places and reach this valuable resource, the compost, and the worms that were at the focus of their investigations. Students had small shovels, hand lenses, spoons, rulers, and petri dishes available on the table for their use. Martine explained that students could move as they wished among the other tables set up around the room–a reading corner that contained both informational and fiction books about worms, or a model of a worm on the center carpet. The digital microscope station was set up on the compost table. The microscope was hooked up to a laptop via a USB cable and sat at one end. Martine stayed at this station as the students worked with the compost so that she could support their first use with the microscope. As she sat, a student stood waiting next to her, his hands cradling a petri dish. *I found a cocoon*, he said in Luxembourgish, turning to face Martine, and presented her with the petri dish. *Okay, let’s see,* she responded*. I am not sure what you found, but we will see*, she replied as she focused on the laptop and opened the required computer program. *I will turn this (microscope) on and open this program*, she said, explaining in Luxembourgish what she was doing to set up the computer. She then said to the table of students investigating in general, *when you have found something, you can come and look (with the microscope)*. She picked up the digital microscope and positioned it over the petri dish the student has brought to her. She looked at the computer screen, and as she adjusted the focus knob on the microscope she let out an, *Ahhhh, (inhaling) Wow! a*s the magnified view came into focus. The student, looking at the same computer screen leaned his head in toward Martine and when he saw what she saw, leaned over closer to Martine and the computer screen, and exclaimed loudly, *Wow*! (Fig. 1, left). As both leaned in toward each other, gazing at the screen Martine said, *it’s really nice, a baby (worm)*, as she inched the microscope lens along the top of the petri dish in order to keep the moving baby worm in the field of vision. Martine had explained in a prior discussion that this student had been interacting and participating in class yet had not verbally participated using Luxembourgish.

A second student heard the emotional exclamations and leaned in for a closer look (Fig. 1, middle) *Wow! Super!* Martine declared again, while looking at the screen and the cocoon. The student, filled with excitement, jumped up and down and pointed at the cocoon on the screen (Fig. 1, right). Martine explained, *It is a baby, you are right*, looking at him and speaking, then turned her gaze back to the laptop screen and the projection from the microscope. *Come, we’ll take a photo* Martine explained, and captured a photo of the cocoon with the microscope photo-capture function. Later, Martin hung these photos around the classroom and referred to them in group discussions about what the students had found. Both students move over to the far end of the table, and returned a few minutes later with two new discoveries, a baby worm, another cocoon. Martine and the student continued looking together. Turner (2012) explains this coming together as a “sense felt by a plurality of people without boundaries” (p.1) and in this case the students directed the coming together to view the cocoon in the space set up by Martine. In this way there is an “inversion of the structural order and the abandonment of status and acquisition” (Turner 2012, p. 9). It is the students who drove the investigation, as Martine supported the investigation of findings presented by the student.

**Fig. 2:**
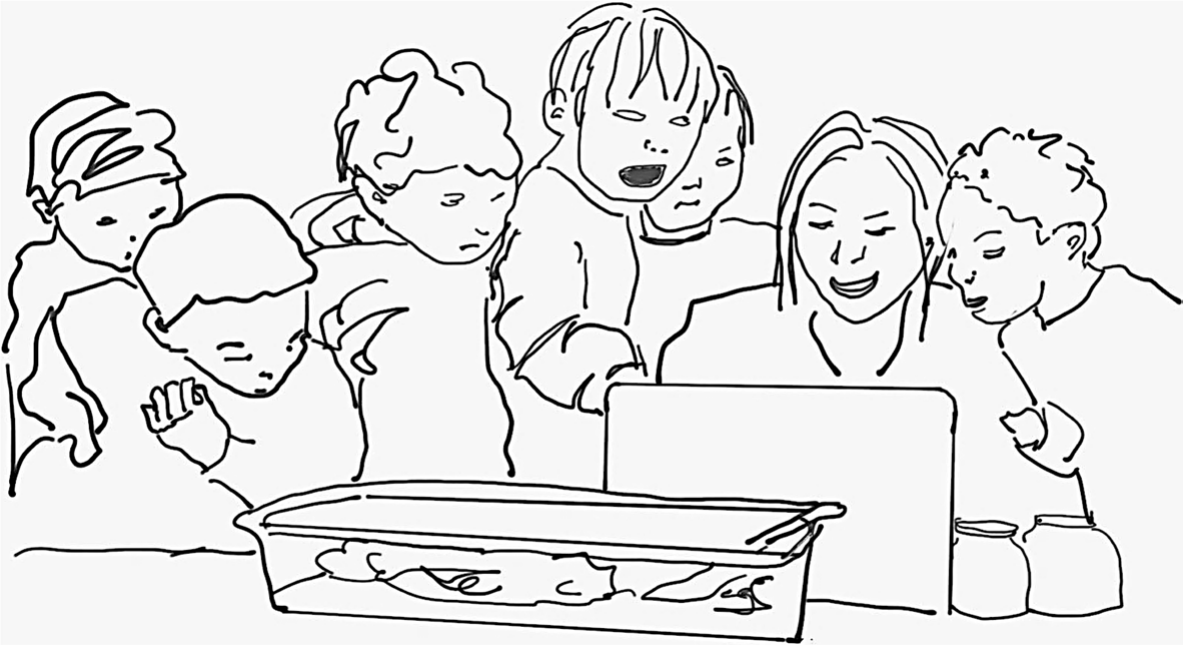
Martine and the students crowd together to observe during a collective moment of intense emotion

A short time later Martine sat at the same station and used the microscope to observe a worm moving in a petri dish. She exclaimed, *The worm made caca*! *Who would like to see*? extending an invitation to the students at the table to come and to look. They rush over in excitement and leaned over, packing in to see the images on the laptop screen (Fig. 2).

As they looked at the laptop screen Martine explained in Luxembourgish, *the worm eats the dirt through its mouth, then through its belly, and it comes out as caca*. The students crowded in to view the laptop screen, as they listened to Martine explaining, observing the moving worm with the dirt in its stomach cavity. Martine continued to explain the parts of the worm they could observe. *Can you tell which end is the head?* As they offer their ideas, Martine pointed to features on the screen. Then, she turned to look at the students to speak. They looked at her, then moved their gazes to look at the laptop screen. There was visible excitement in these moments. These are student-found treasures—the cocoon, the baby worm, and treasures found by Martine that border on the silly—the caca–the students' movements, exclamations, bodies, and gaze toward the computer reveal their collective intrigue and joy, they are excited by these new worlds that they can see with the microscope. Martine designed this space to be open, to allow students to come and go, to have choice in their science investigations. She facilitated the use of the microscope, and let their interests guide the investigations. With her tone, with her verbal invitations, and by sitting with them, together they decided what to explore. Joy developed through their collective exploration.

### Vignette 2: That is my hair!

**Fig. 3:**
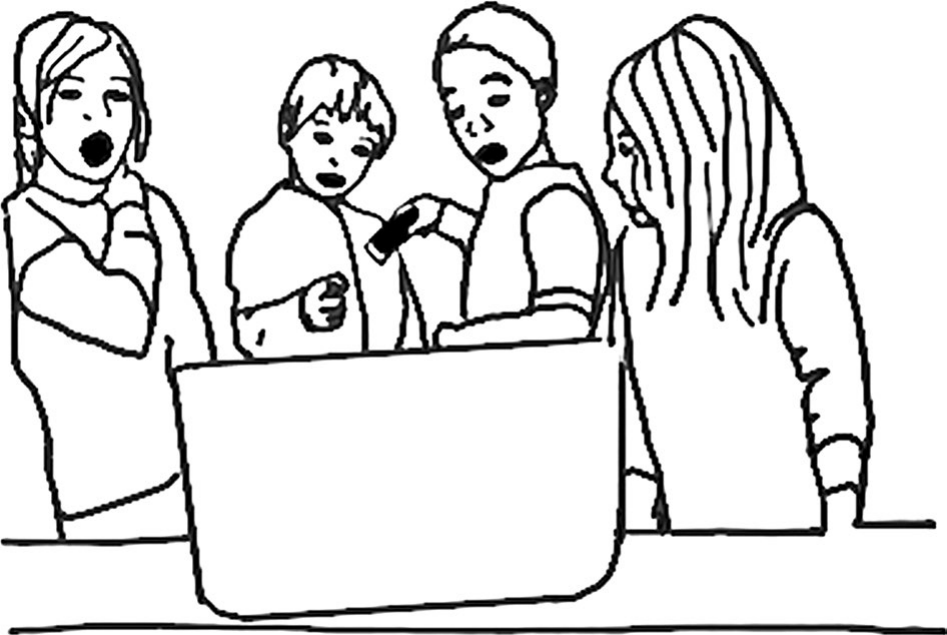
Collective joy and surprise at observing magnified fleece fabric with a digital microscope

At a later point during the Worms and Compost project, Martine setup the microscope station for students to use independently. At one point a student stood at the microscope station alone. She looked at her fingers. Then, she used the photo-capture function on the microscope to capture a screen shot of the image projected on the laptop screen. Other students joined her. Together they focused their gazes on the laptop screen. Over a period of several minutes, three students gathered. They looked at their fingers, up their noses. *Take a photo*, the first student prompted in Luxembourgish after pointing at the computer and showing the group the images she captured. The second student took a photo. *Bravo!* she encouraged. They continued looking together, in her mouth, at his fingers. A fourth student joined in the collective looking. He placed the microscope on his head, *My hair*! he shouted as he saw this part of himself up-close and magnified for the first time. *That is my hair!* he exclaimed pointing to the image of his hair displayed on the screen. A fourth student had been working nearby, and as often happens with open spaces of exploration, was drawn in by the emotional excitement. The fourth student walked up to the group and leaned over to look at the laptop screen. *What should we look at?* asked the fourth student? *My eye*, responded the third. *Take a photo*, he prompted. A photo was taken, and the third student exclaimed *Yea!* and jumped up and down with joy. They passed the microscope among themselves. They looked at one another’s’ hair. With each new item they observed, they would togther focus their gaze at the person who was being examined, then turn to the laptop screen to see the magnified image, shifting from viewing with their eyes, to viewing with the microscope, back and forth. This repeated ritual looking was punctuated with moments of expressed emotion. Shock! Excitement! Surprise! They continued and next observed each other’s shirt fabrics. A cotton sweatshirt. A blue fleece jacket. They turned their heads toward the magnified view of the blue fleece and a collective *Woooooooow!* arose from the group (Fig. 3). Their mouths in “O”s of astonishment. Science here is group exploration and observation, a passing of the microscope from one to the other, looking at you, looking at me. What was observed was chosen by the members of the group, each contributed to the exploration. Turner (2012) explains this contribution of the individual to the collective during *communitas* as collective while supporting the individual, “It does not merge identities; the gifts of each and every person are alive to the fullest. It remains a spring of pure possibility, and it finds oneness, in surprise” (p.3) which develops during “collective tasks with full attention” (p.3).

## Collective joy and *communitas* in observation

What I have showed through these two vignettes is that an examination of students’ and teachers’ interactions on micro-levels when viewed through the lens of *communitas*, provides a beautiful view into the collective emotions that arose as students worked with digital microscopes to investigate their world. Collective looking and emotional engagement occurred during both teacher-guided exploration (Vignette 1) and during student-guided exploration (Vignette 2). As they worked with the digital microscope, interaction rituals formed that allowed for the buildup of positive emotional energy (EE) in ways that formed the basis for closeness, bonding, and collective looking. Through interactions, students collectively experienced wonderment, joy, excitement, and pride. Through participation in science, and in observing, they were able to experience *communitas*, a space of collective joy in their exploration with the digital microscopes.

In both vignettes the social order was fluid relative to other spaces in the classroom: the teacher and students looked together and experienced the wonderment of seeing a worm cocoon structure up close for the first time. In this space, the difference in power between teacher a student was bridged. All students participated, and none was dominant as the microscope was passed from hand-to-hand. Ideas flowed and the focus of their observation shifted fluidly. Language, which in many spaces in our national education acts as a gatekeeper to participation in science instruction (Wilmes et al., 2018), became one of many resources students drew upon to engage in science. This was not a space where one student spoke a better form of Luxembourgish than another, all were welcome to participate, and the emotions produced were collective, embodied, and contagious. Interaction ritual analysis allowed us to see the emotional geographies that developed in this learning space. We see how the bodies that interacted with science materials moved together, apart, toward one another with heads bowed toward the laptop screen, with mouths open in collective exasperation, development of collective joy through investigation revealed.

Analysis of micro-level interactions showed that the instructional approaches Martine set up allowed the space for students to work with her, and with each other, and to build positive emotional experiences as they engaged in science practices over time. This is an important emotional facet of early childhood classrooms in which students are first experiencing “formal” science education. Their first sciences experiences are within spaces that allow them to bond with one other, to experience wonder and joy together. Their first experiences with science are joyful and collective. They draw upon diverse resources and through doing so are able to participate in and through their bodies and emotions.

Additionally of note, is that science investigations in Martine’s classroom were driven by students' interests in a provided theme. This underscores the power of grounding science instruction in students' questions and interests as a transgressive practice. As Siry and Brendel (2016) explain,

When children are given the space to pursue their wonderings and questions; individually or collectively, in classrooms or informal settings, guided or free. The boundaries that are set by structures such as science curriculum policy are porous, and a focus on enjoyment, and ideally on happiness, can serve as an approach to transgress such boundaries. (p.11)

These spaces where children can direct investigations based on their curiosities can be places where communitas forms, and science manifests through collective joy.

## Implications for research and teaching

Implications arise from this work for both research and teaching. The findings illustrated support views of science teaching and learning as embodied and material practices. They point the way to further research, grounded in embodied multimodal views of interaction and communication, that illuminate the great diversity of resources students draw upon to engage in science. Theoretical and methodological lenses that allow us to “see” and “hear” these embodied resources provide much richer views of students’ engagement and thus can be further employed to examine students’ engagement in science practices. As I have shown here, and as multiple studies from our research group have illustrated, this is especially key for students who are learning a language as they also learn to science (Siry and Gorges 2020). An examination of classroom practices through interaction ritual analysis helps us as teachers come to understand how students can be provided with space to engage diverse communicative and interactional resources, and to see a way forward to supporting participation in school science. Open spaces of science investigation, that allow her students to drive explorations, allow not only for engagement in science practices regardless of students’ language proficiency, but for full participation and the development of collective joy.

## Concluding thoughts

I write this manuscript at a time when the COVID-19 pandemic has wreaked havoc on our collective lives. The future form of collective life within classrooms has come into question. Our communities have been ripped apart from our assumed ways of being and doing, and we have been slammed together in new ways –digitally and over virtual distances—that require us to bond with one another often from intimate home-like spaces, yet over greater distances. As I reflect upon the collective spaces that Martine created in her classroom and with her students last winter, the joy, the confidence, and the *communitas* that developed among them, juxtaposed with the current educational struggle to continue through virtual modes, my feelings are overwhelming. I write this forum article to celebrate these joyful spaces. I write this forum so that we remember what *was* possible in a classroom where students could lean in together in close proximity and bond: this may not happen again any time soon. This was a classroom where difference was positioned as a resource (Siry 2011), and status did not matter in the moment, because collective *science* was happening. Students were immersed in this process of science as communitas: collective joy. I write this to remember these moments. I write this to inspire future moments.

## References

[CR1] Baroni P, Cadenelli N, Caprara B, Colombi A, Fogli D, Scala C, Serina I (2014). On the use of digital microscopes at nursery and primary schools. Procedia-Social and Behavioral Sciences.

[CR2] Collins R (2004). Interaction Ritual Chains.

[CR4] Council of Europe (2018). Common European framework of reference for languages: Learning, teaching, assessment. Companion volume with new descriptors. Strasbourg: Council of Europe. Available at https://www.coe.int/en/web/common-european-framework-reference-languages

[CR5] Hubbard, P. (2007). The geographies of ‘going out’: Emotion and embodiment in the evening economy. In J. Davidson, L. Bondi & M. Smith (Eds.) *Emotional Geographies* (pp. 117–134).

[CR6] Kinsley M, Capraro K (2013). Small wonders, close encounters: Introducing students to the world of microscopy. Science and Children.

[CR20] Lupton D (1998). The emotional self: A sociocultural exploration.

[CR7] Ministère de l’Éducation nationale et de la Formation professionnelle (MENFP). (2011). École fondamentale. Plan d’études. Luxembourg. Available online https://men.public.lu/fr/publications/courriers-education-nationale/numeros-speciaux/plan-etudes-ecoles-fondamentale.html.

[CR8] Ministère de l’Éducation nationale, de l’Enfance et de la Jeunesse (MENJE. (2019). Statistiques globales et analyse des résultats scolaires - Enseignement fondamental - 2016–2018. Available online https://men.public.lu/fr/publications/statistiques-etudes/fondamental/2011-ef-statistiques-globales-2018-2019.html.

[CR9] Milne C, Otieno T (2007). Understanding engagement: Science demonstrations and emotional energy. Science Education.

[CR10] Olitsky S (2007). Promoting student engagement in science: Interaction rituals and the pursuit of a community of practice. Journal of Research in Science Teaching.

[CR50] Olitsky S (2021). Identity, agency, and the internal conversations of science and math teachers implementing instructional reforms in high-need urban schools. Cultural Studies of Science Education.

[CR22] Oxford Latin Dictionary. (1982). P. G. W. Glare (Ed.). Oxford: Oxford University Press.

[CR12] Rinchen S, Ritchie SM, Bellocchi A (2016). Emotional climate of a pre-service science teacher education class in Bhutan. Cultural Studies of Science Education.

[CR24] Siry C (2011). Exploring the significance of resource-rich views in science education. Cultural Studies of Science Education.

[CR13] Siry, C., Andersen, K. N., & Wilmes, S. (2018). „Doing Science “: Erwerb von Kompetenzen im naturwissenschaftlichen Unterricht der École fondamentale. Nationaler Bildungsbericht Luxemburg 2018, 140-141. Available online https://www.bildungsbericht.lu/multilingualitaet-unterricht/.

[CR14] Siry C, Brendel M (2016). The inseparable role of emotions in the teaching and learning of primary school science. Cultural Studies of Science Education.

[CR23] Siry C, Gorges A (2020). Young students’ diverse resources for meaning making in science: learning from multilingual contexts. International Journal of Science Education.

[CR15] Siry C, Kremer I (2011). Children explain the rainbow: Using young children’s ideas to guide science curricula. Journal of Science Education and Technology.

[CR17] Turner V (1969). The ritual process: Structure and anti-structure.

[CR16] Turner E (2012). Communitas: The Anthropology of collective joy.

[CR18] Wilmes SED, Siry C (2018). Interaction rituals and inquiry-based science instruction: Analysis of student participation in small-group investigations in a multilingual classroom. Science education.

[CR21] Wilmes SE, Siry C (2021). Multimodal Interaction Analysis: a powerful tool for examining plurilingualstudents’ engagement in science practices. Research in Science Education.

[CR19] Wilmes SED, Siry C, Gómez Fernández R, Gorges A, Bryan LA, Tobin K (2018). Reconstructing science education within the language | science relationship: Reflections from multilingual contexts. 13 Questions. Reframing education's conversation: Science.

